# Implementation of a Depth from Light Field Algorithm on FPGA

**DOI:** 10.3390/s19163562

**Published:** 2019-08-15

**Authors:** Cristina Domínguez Conde, Jonas Philipp Lüke, Fernando Rosa González

**Affiliations:** Departamento de Ingeniería Industrial, Universidad de La Laguna, Apartado 456, 38200 La Laguna, S/C de Tenerife, Spain

**Keywords:** light field, disparity estimation, FPGA

## Abstract

A light field is a four-dimensional function that grabs the intensity of light rays traversing an empty space at each point. The light field can be captured using devices designed specifically for this purpose and it allows one to extract depth information about the scene. Most light-field algorithms require a huge amount of processing power. Fortunately, in recent years, parallel hardware has evolved and enables such volumes of data to be processed. Field programmable gate arrays are one such option. In this paper, we propose two hardware designs that share a common construction block to compute a disparity map from light-field data. The first design employs serial data input into the hardware, while the second employs view parallel input. These designs focus on performing calculations during data read-in and producing results only a few clock cycles after read-in. Several experiments were conducted. First, the influence of using fixed-point arithmetic on accuracy was tested using synthetic light-field data. Also tests on actual light field data were performed. The performance was compared to that of a CPU, as well as an embedded processor. Our designs showed similar performance to the former and outperformed the latter. For further comparison, we also discuss the performance difference between our designs and other designs described in the literature.

## 1. Introduction

### 1.1. The Light Field and How It Encodes Depth

**Figure 1:**
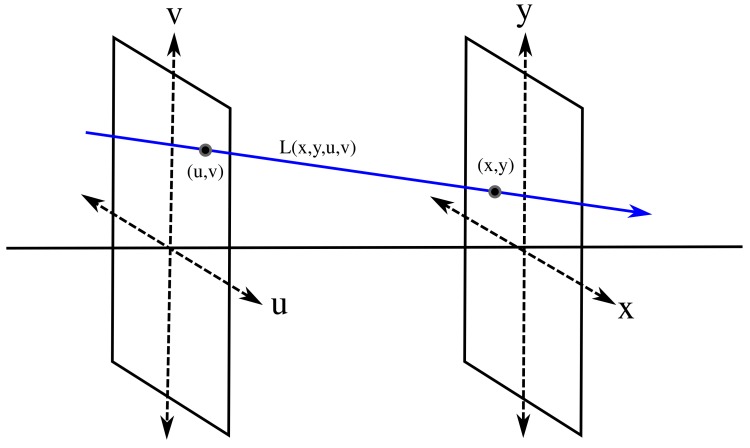
Two-plane parameterization of a light field.

The light field is a 4D function that grabs the light information in a scene. It is a reduced version of the plenoptic function proposed in [1]. The plenoptic function is a 7D function parameterized by $\left(V_{x},V_{y},V_{z},\theta ,\phi ,\lambda ,t\right)$, where $\left(V_{x},V_{y},V_{z}\right)$ is a point in 3D space, (θ,ϕ) are the incidence angles of a ray traversing that point, λ is the wavelength of light and *t* represents time. This representation models the light in a scene. In [2] and [3], it was proposed to reduce the plenoptic function to four dimensions for the case of light in free space. This reduction is based on two assumptions: first, we can omit *t* and λ to simplify the notation, and, second, the radiance along a line in free space is constant, so one only needs to parameterize that line instead of all points on it. The reduced version can be parameterized in several ways, but the two-plane parameterization is the most common. The light field L(x,y,u,v) grabs the intensity of rays in free space. To parameterize the rays, two parallel reference planes are used, as shown in Figure 1. The cut point with the u−v plane and the cut point with the x−y plane define a ray. Varying the coordinates (x,y) while maintaining (u,v) as fixed defines a conventional 2D image from a certain viewpoint. On the other hand, fixing (x,y) and varying (u,v) retrieves the intensity of all the rays impinging on the point (x,y) from different directions parameterized by (u,v). As the acquisition is done with a digital device, each dimension captures a discrete set of data. The number of pixels in the *x* and *y* dimensions are given by $N_{x}$ and $N_{y}$ respectively, while the number of viewpoints in (u,v) is $N_{uv}\times N_{uv}$.

The light field encodes depth information in the structure of intensity values [4]. In the case of a Lambertian scene (no intensity change with direction), the depth for each point relates to all views in the following way:

(1) L(x+su,y+sv,u,v)=c,∀(u,v)

where *c* is the constant intensity of the point and *s* is the disparity. With this information at hand, the extraction of depth information converts into detecting this structure or, in other words, into the problem of estimating *s* from a set of views (u,v). In a later stage, the depth *z* can be obtained using the following expression:

(2) $$z=\frac{1}{\alpha +\beta s}$$

where α and β are calibration parameters.

### 1.2. The Light-Field Pipeline

**Figure 2:**
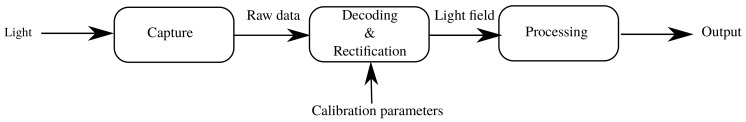
General light-field pipeline

To capture real light-field data and use them in an application, the general pipeline depicted in Figure 2 must be followed. First, raw light-field data are captured. After that, the raw data must be decoded and rectified using the calibration data as input and then, finally, they can be processed to obtain a useful data product. The following sections describe each of the stages of the pipeline and analyse the different approaches described in the literature.

#### 1.2.1. Capture

The light field is a mathematical concept that describes the light in a scene. To capture real light-field data, several techniques can be used [5]. Most of them consist of taking images from different viewpoints using an optical setup. In general, four categories of capture methods are distinguished:

- Camera arrays: This technique consists of placing a set of cameras on a plane at equally spaced distances. Examples of this capture technique can be found in [6,7]. These kinds of devices were initially considered to be very bulky and difficult to operate, but with the advent of smart phones, the size of cameras has been considerably reduced and a portable camera array design is not so unrealistic.
- Plenoptic cameras (spatial multiplexing): The plenoptic camera is the most well known light-field capturing device [8,9,10,11]. It consists of a microlens array placed between the main lens and the sensor of a conventional camera. The microlenses spread the spatial information and the angular information contained in the light field over a 2D sensor. The main advantage is the compactness, since it is similar in size to a conventional camera. However, it has the drawback of having to trade-off between spatial resolution and angular resolution, because both are spread over the same sensor [12]. This means that the spatial resolution is reduced in comparison to conventional cameras.
- Temporal multiplexing: This technique consists of one camera that moves to different positions over time using a robotic arm or a gantry [2,13]. The main drawback of this approach is that it works only for static scenes.
- Frequency multiplexing and compressive sensing: This approach uses a single camera with an optical mask [14,15,16,17]. The raw data must then be decoded using a computationally expensive process to obtain a light field.

#### 1.2.2. Decoding and Rectification

Once raw light-field data have been captured, they must be decoded and rectified to provide adequate input for the processing algorithms. This process includes tasks like colour demosaicing, denoising and others that improve data before further processing [18,19,20,21,22,23,24].

#### 1.2.3. Processing

The decoding and rectification produce a properly parameterized light field. The light field can then be processed with different techniques to obtain different output data products. The processing algorithms can be classified into different categories depending on the output they generate. In the bullets that follow, we describe the different types:

- Light-field rendering: This type of algorithm computes a new 2D image or a set of such images from light-field data. Isaksen et al. [25] proposed a reparameterization to generate images on a general surface from light fields. However, the most prominent algorithm in this category, which is used for the computation of the focal stack, was proposed in [26]. The main drawback of this focal-stack algorithm is the low spatial resolution of the outcoming images. To overcome this drawback, superresolution algorithms were proposed [27,28,29,30,31,32]. Other authors have also proposed techniques to accelerate the computation or to make it more accurate [33,34].
- Depth from light field: The goal of depth from light field algorithms is to extract depth information from the light field and provide a depth or disparity map that can be sparse or dense. Several approaches can be adopted to perform this processing task. One strategy is to convert the light field into a focal stack (that is, a set of images focused at different depths) using light-field rendering techniques and then estimate depth by applying a depth from focus algorithm [35,36,37]. Another group of techniques is based on the computation of the variance focal stack to extract depth information [28,34,38]. Berent and Dragotti [39] decided to use image segmentation techniques on light fields to detect the plenoptic structures that encode the depth information, as mentioned in Section 1.1. Other authors use differential operators to detect the slope of plenoptic structures [8,40,41,42]. In [43], robust PCA is used to extract depth information from plenoptic structures. On the other hand, Kim et al. [44] developed a depth estimator for high spatial angular resolution light fields, while Jeon et al. [45] present an accurate method for depth estimation from plenoptic camera. In [46], the authors proposed a pipeline that automatically determines the best configuration for the photo-consistency measure using a learning-based framework. Recently, the use of convolutional neural networks to estimate depths from a light field has been investigated [47,48,49].
- Wavefront sensing: The purpose of this approach is to extract an estimation of the wavefront phase in the scene. This technique has been widely explored in astrophyshics to perform adaptive optics in telescopes [50,51,52,53,54].
- Tracking and pose estimation: As the light field encodes 3D information about the scene, it can be used in visual odometry and navigation. In [55], theoretical developments to extract pose information from light fields were presented. In [56], some algorithms for visual odometry were given. Further explorations in this application field can be found in [57,58,59]. Also some studies focused on using the technology in space applications [60,61]. The light field features in scale space and depth proposed in [62] can also be included in this category of algorithms.

### 1.3. Light-Field Processing on FPGA

The main issue with light field processing algorithms is the enormous amount of data that must be processed. Each captured light field has a shape of $N_{x}\times N_{y}\times N_{uv}\times N_{uv}$. For instance, the light fields used by [26] have a shape of 292×292×13×13, which is about 15 megarays. To achieve real-time performance, parallel or specific hardware architectures must be used. The most common choice in this case has been GPUs (Graphics Processing Units). The implementation of light-field algorithms on an embedded system is very difficult when real-time performance needs to be reached. In recent years, the trend has been to make use of SoC (System on Chip) approach, which integrates all parts of a computation system on a single chip. Most often, a SoC is composed of a CPU and specialised processing units such as GPUs, MPUs and DSPs. These are are surrounded by other peripherals, such as memories, GPIOs or buses. It is also possible to implement custom hardware components in reprogrammable devices, such as FPGAs (Field-programmable gate arrays). FPGA manufacturers have their own product lines consisting of programmable hardware that is connected to a CPU and other peripherals [63,64]. This configuration allows system designers to decide which parts of the system should run on the CPU and which should be outsourced to a hardware block via a hardware-software co-design.

Only a few examples of light-field processing algorithms on FPGA exist in the literature. Some light-field rendering algorithms have been implemented [65,66,67], and also some effort has been made to implement wavefront phase estimation in real time [68,69]. One of the first examples of depth from light field on FPGA can be found in [70], where the authors proposed an architecture that implements the belief propagation algorithm and used it on light-field data. On the other hand, Chang et al. [71] provided a pixel-based implementation of a depth from light field algorithm. They used a modified raster scan and pipelining to improve the performance of their implementation.

### 1.4. Goals and Contributions of This Paper

This paper addresses the design and implementation of a depth from light field algorithm on FPGA. Data acquisition, decoding and rectification of light-field data are not in the scope of this paper. In a more concrete fashion, we show the design of hardware components that run on FPGA to process the incoming light fields and generate a disparity map as an output. Two sources of light-field data are considered. The first employs input data provided by a plenoptic camera, and the second employs input data provided by a camera array. Other input data sources were not considered because temporal multiplexing is only suitable for static scenes and frequency multiplexing and compressive sensing need a decoding step that is computationally heavy.

The contributions of this paper are two light field data processing architectures (one for each input format) based on a common basic processing block. An array of such blocks is surrounded with the control and adaptor logic needed to process each input format. Both architectures can process a light field during read-in and can produce a disparity map only a few clock cycles after the last pixel is read in. Other architectures need a loading step to be completed before data can be processed. Furthermore, with this approach the use of on-chip memory is reduced substantially in our designs compared to other depth from light field designs on FPGA and it is not necessary to use off-chip memory. Finally, the performance of each architecture is tested and discussed.

## 2. Implementation

### 2.1. The Algorithm

In this paper, we focus on local estimators because they are especially well-suited for implementation on FPGA. These estimators aim to compute the depth for a point using a local neighbourhood. Most local algorithms perform the estimation based on the gradient of the light field [8,40,41,42], which makes this is a necessary previous step to estimate the disparity *s*. The light-field gradients here are estimated using the method proposed by Farid and Simoncelli in [72], although other derivative filters could be used. Farid and Simoncelli [72] proposed a set of optimised and separable derivative filters for multidimensional signals. The 3-tap filter is composed of the two 1D separable filters shown in Equation (3). The *d* filter is applied in the direction of the partial derivative, while the *p* filter is applied in the other directions. Remember that a light field is a 4D signal, which means the derivatives will be obtained by convolving with *d* in the direction of the derivative and convolving with *p* in the other three directions. Of course, filter sizes other than 3 can be used.

(3) $$\begin{array}{c} \vec{p}\;=\left(\;\;0.229879\;\;0.540242\;\;0.229879\right)\\ \vec{d}\;=\left(-0.425287\;\;0.000000\;\;0.425287\right) \end{array}$$

Once the derivatives along the four dimensions of the light field have been computed, a gradient vector $\left(L_{x},L_{y},L_{u},L_{v}\right)$ can be composed. Now, to obtain an estimation of the disparity, [8] proposes using the expression of Equation (4).

(4) $$s=\frac{\sum_{P}L_{x}L_{u}+L_{y}L_{v}}{\sum_{P}L_{x}^{2}+L_{y}^{2}}$$

where *P* is a 4D patch around the pixel for which we are computing the disparity. In this paper, we reduce the cardinality of *P* to 1. We must note that other methods can be used to compute disparity from derivatives, but they would require more resources on FPGA as they need trigonometric operations [41,42].

Algorithm 1 shows the general structure of the algorithm to be implemented. Please note that Step 1 is a 4D convolution that can be implemented using the separability of the filters, but we do not exploit this approach here, as we only need 3-tap filters and symmetry allows us to reduce the number of operations, as we explain later. The use of 3-tap filters makes *h* equal to 1, since the filtering cannot be performed at the borders and the variables l,j,k,n are constrained to the set {−1,0,1}. Furthermore, we restrict our study to 3×3 views ($N_{uv}=3$). This means we only will obtain a result for the central image of the light field (u=1, v=1). Step 2 of the algorithm computes disparity values from the gradients computed in Step 1 using Equation (4).
**Algorithm 1:** Local depth from light-field algorithm.
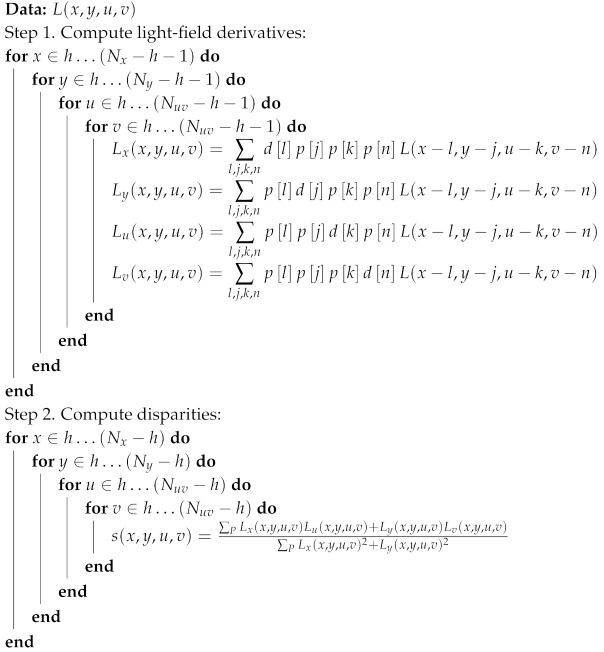


### 2.2. System Architecture

**Figure 3:**
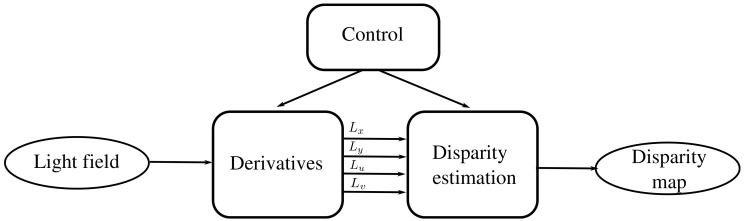
General system architecture.

The system to process the light field into a disparity map includes the three modules presented in Figure 3. The mission of the derivative module is to compute the partial derivatives of the light field taken as input. The derivatives $\left(L_{x},L_{y},L_{u},L_{v}\right)$ are then fed into a disparity estimation module that computes a disparity value for each pixel of the light field’s central view. Each module must take the data when they are available and produces an output only when a partial result can be given. The process is controlled by a control module that generates the pertinent control signals fed to each module. Fixed-point arithmetic is used (16 bits for the integer part and 16 for the fractional part).

The light-field data can have different formats depending on the device used for capture or other processing steps. In the next section, we discuss two different data formats.

### 2.3. Data Input Format Considerations

The decoding and rectification algorithm in the light-field pipeline generates a stream of pixels corresponding to the intensity of the rays in the light field. However, pixels cannot be received in parallel by a processing module. In addition, pixel ordering may be different depending on the type of capturing device used. The processor must wait to receive the data needed to generate a certain output pixel. The waiting time to generate one output pixel can be used to calculate partial results for other output pixels, but this requires an intermediate memory to store partial results. Due to this requirement, the timing and ordering of the input data stream strongly conditions the processor design.

Here two input formats are considered. First, we considered the data produced by a plenoptic camera. In that case, pixels are read in a sequential manner and the ordering of the sequence is given by $i=y\cdot N_{x}\cdot N_{uv}\cdot N_{uv}+v\cdot N_{x}\cdot N_{uv}+x\cdot N_{uv}+u$. Second, we considered input data provided by a camera array. In that case, $N_{uv}\times N_{uv}$ pixels corresponding to the position (x,y) can be read-out in parallel, at each clock pulse. The pixel order is given by $i=y\cdot N_{x}+x$. We call the first input format *serial input* and the second input format *view parallel input*.

As the first output pixel can only be produced when all the input pixels necessary to produce it are available, the time instants and speed of the calculation depend on the input format. We implemented both of our two input formats assuming that the light field comprises 3×3 views ($N_{uv}=3$), although this could be changed in the future.

#### 2.3.1. Serial Input

As the name implies, serial input data arrive serially, which means that not all pixels used to produce an output are available at the same time. Here we explore the possibility of performing the convolutions shown in Algorithm 1, calculating partial results as the data arrive. At each clock cycle, only one pixel is available, but it still contributes to a set of outputs. Given a ray (x,y,u,v) the positions of the derivatives it contributes to are those in Equation (5).

(5) {(x+l,y+j,u+k,v+n)|l,j,k,n∈{−1,0,1}}

Please note that the indices *l*, *j*, *k* and *n* are in {−1,0,1} because our implementation uses 3-tap filters. Furthermore, if the intention is to perform the computation only for the central view, in the case of $N_{uv}=3$, the set in Equation (5) reduces to the set shown in Equation (6).

(6) {(x+l,y+j,1,1)|l,j∈{−1,0,1}}

Equation (5) means that each input pixel in the light field contributes to a 3×3 neighbourhood around its position (x,y) in the central view. In terms of code, this means that each time a ray arrives, the actions shown in Algorithm 2 must be performed.
**Algorithm 2:** Computation of partial result for a given input pixel
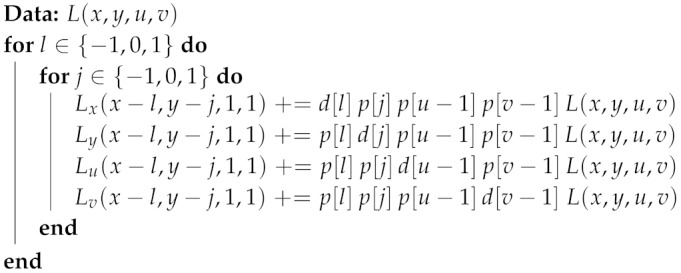


Algorithm 2 allows one to make the following interesting observations:

- The four derivatives can be performed in parallel.
- The ‘for’ loops may be performed in parallel as there is no dependency between iterations.
- Each time a pixel arrives, its product with a subset of 9 of 81 possible filter coefficients must be calculated.

In exploring the symmetry and zeros in the filter coefficients, some simplifications can be carried out. Please note that p[−1]=p[1], d[1]=−d[−1] and d[0]=0. If the sign change in *d* is applied later, then the possible non-zero values for the coefficients reduce to the four values listed in Table 1.

**Figure 4:**
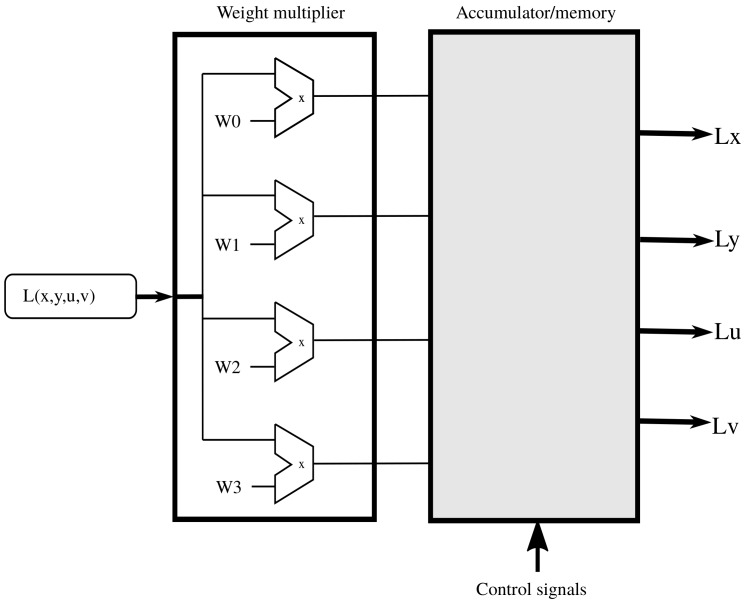
Implementation of weight multipliers for serial input.

Each time a pixel comes into the system, the four values are multiplied by the pixel value. After that, we still must decide where to add each product and if a change of sign must be performed. Figure 4 shows the schematic diagram of the hardware implementation used to process one pixel. The pixel goes into the *weight multiplier* block where the pixel value is multiplied by each of the four non-zero weights mentioned before. The signs of the four values might change before they are added to the corresponding partial result, which happens when they are fed into the accumulator-memory. We describe the latter in Section 2.4.

#### 2.3.2. View Parallel Input

**Figure 5:**
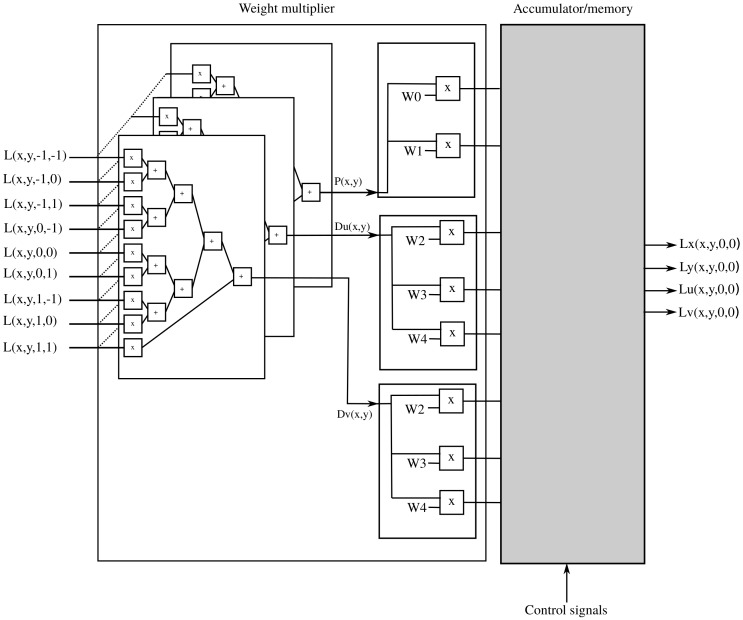
Weight multiplier for view parallel input.

With the view parallel input format, the 3×3 rays corresponding to a pixel position (x,y) are available at the same time. This allows us to adapt Algorithm 2 to get Algorithm 3, whose hardware implementation is shown in Figure 5. First, the product of the 3×3 values coming in with the adequate filter coefficients is calculated in parallel. We then use a parallel adder tree with pipelined architecture to get $D_{u}\left(x,y\right)$, $D_{v}\left(x,y\right)$ and P(x,y). Then those values are multiplied by the coefficients in Table 2, which were obtained using symmetry conditions in a similar manner as in the previous section. Please note that this approach only works with filters that are separable in (x,y) - (u,v). This process results in eight values that are fed into the accumulator memory, whose implementation we show in Section 2.4.
**Algorithm 3:** Computation of partial result for a given parallel input pixel set.
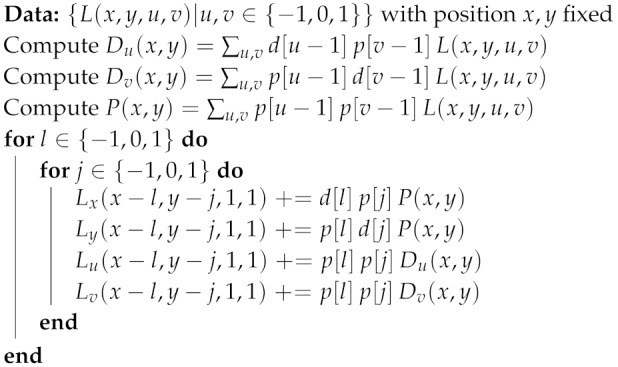


### 2.4. Accumulator Memory Grid

#### 2.4.1. Basic Component

We first consider what is needed to get one of the partial derivatives at position (x,y) in the central view. The obvious solution is an accumulator that adds the right value when arriving. In addition to the accumulator, a module that decides which of the incoming values must be added (and inverted if necessary) must be developed. Following with that strategy, to compute the derivative for all the pixel positions, such a hardware device must be replicated as much times as pixels are in the light field. However, it is also clear that each time a pixel arrives, only 3×3 devices will be active because the incoming values only affect their neighbour pixels rather than all pixels. Replacing the accumulator with an accumulator with a memory component that can store partial results that are not currently affected by the incoming value allows one to create a hardware architecture which is feasible in practice and does not waste resources.

**Figure 6:**
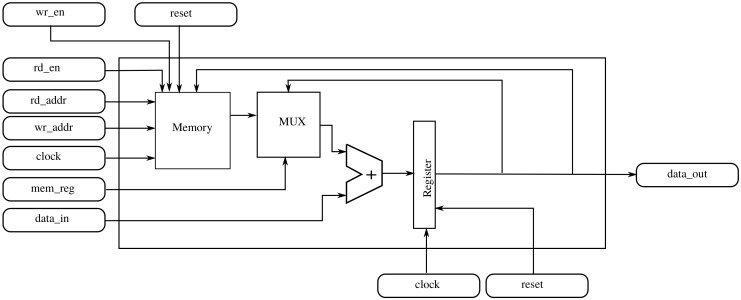
Block diagram of basic component.

Figure 6 shows a detailed block diagram of the basic component, while Table 3 describes the I/O signals. Including a memory allows the reuse of the component across different pixel positions. With this approach, a grid of 3×3 modules corresponding to the nine values updated every clock cycle is needed.

**Figure 7:**
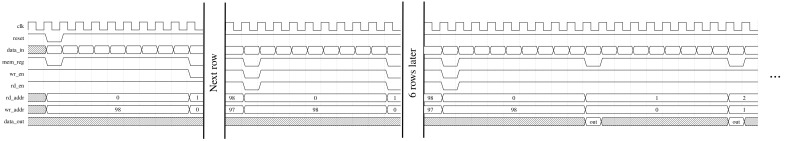
Simplified time diagram for using basic component with serial input.

Figure 7 shows a time diagram that illustrates the working principle of the basic block for a serial input. Please note that here the selection of the right input data has been left out and that we only focus on the process of accumulation and output data generation. Suppose we are computing the derivative of the point (1,1,1,1) in a light field of size (99,99,3,3). When the system starts, the basic component must accumulate all the values that contribute to the derivative at that point. The diagram in Figure 7 shows that, when the first value comes into the component through data_in, the memory is not enabled and its output is zero (rd_en=0 and mem_reg=0). The effect of this configuration is that the value of data_in will go directly to the register. At the second clock cycle, the new value must be accumulated to the register, and nothing must be written to memory (rd_en=1, mem_reg=1, wr_en=1). The same will happen for the other values until the tenth value arrives. This value corresponds to another *x* coordinate, so the partial result in the accumulator must be stored (wr_en=0) and simultaneously the register will be loaded with the value of data_in driving rd_en=1 and mem_reg=0. The partial result for pixel (1,1) will be stored at the memory position of the memory as indicated by wr_addr. This process will repeat for the other values of the same row in the plenoptic image. When the next row begins, it is time to recover the partial result for (1,1) from memory (rd_addr=0, mem_reg=1) and simultaneously the partial result for position 98 will be stored. This process must continue for each pixel of the current row and the other six rows. When the first data value of the last row that contributes to that derivative arrives, the partial result is recovered from memory and nine values are accumulated. After that, the derivative at point (1,1) is in the accumulator and is put into data_out. Nine clock cycles later, a new result is produced by this component for position (1,4) stored at address position 1. (Intermediate pixels will be computed by other components in the grid, as we explain in the next section).

A similar approach can be implemented for the parallel input. In that case, however, the space between the load and storage operations is reduced to every three input values instead of nine.

#### 2.4.2. Basic-Component Grid

**Figure 8:**
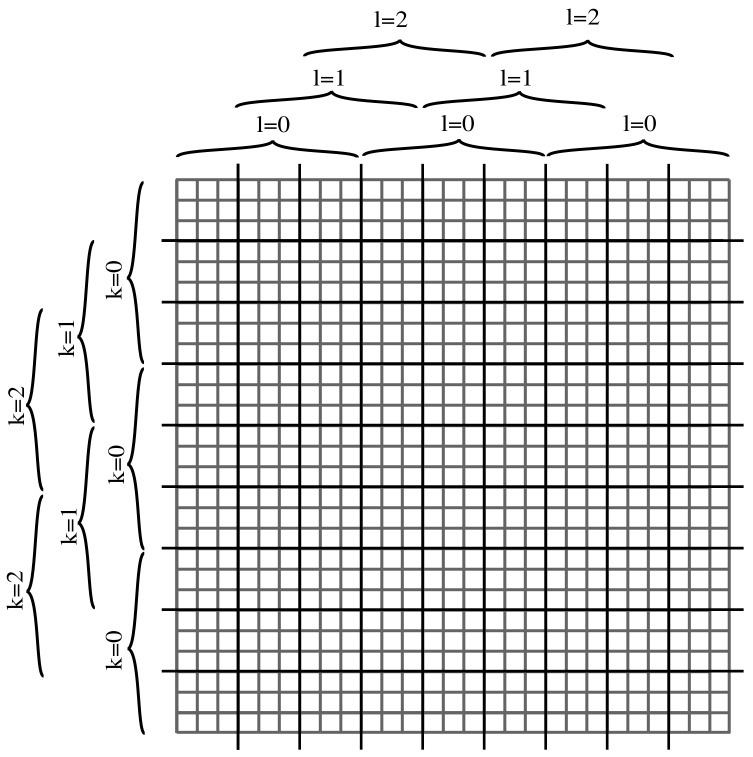
Area of plenoptic image processed by each basic component (l,k).

**Figure 9:**
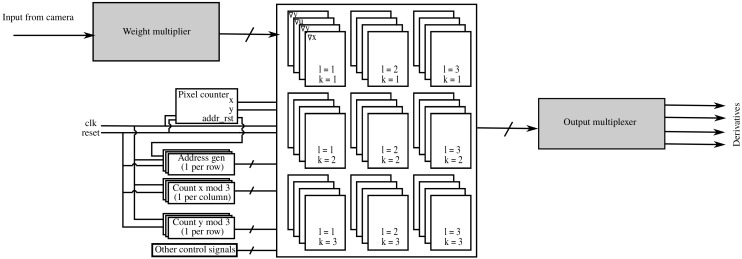
Schematic diagram of grid structure.

With the basic component’s operational approach described in Section 2.4.1, the derivative is computed only at certain positions of the light field. The intermediate positions must be computed by other basic components. These comprise a 3×3 grid indexed as (l,k). Figure 8 shows a light field formatted as a plenoptic image. Each block of the grid is responsible for an area of the light field. Except at border regions, the nine basic components must operate continuously on the incoming data. Furthermore, the grid must be replicated four times, one for each derivative. In each clock cycle, one of the basic components generates an output which must be multiplexed from each individual block to the general output. Figure 9 shows the grid’s general structure.

Up to this point, we have not mentioned the connection between each basic block and the input signals coming from the *weight multiplier* described in Section 2.3.1 for serial input and Section 2.3.2 for view parallel input. Each of the grid’s basic components must be accompanied by a data selector that selects the right ‘data × weight’ for each position and time instant. The decision is made based on a set of modular counters and other control logic, as depicted in Figure 9, that allow each block to decide which input to select. Obviously, the selection method differs depending on the input format (serial or view parallel).

Finally, each block needs to channel its output to the general output. This is done with a multiplexer governed with a control signal that decides which block will be forwarded to the general system’s output.

### 2.5. Disparity Estimation

Once the derivatives have been computed, the disparities can be estimated using different local methods [8,40,41,42]. An output adaptor was designed to estimate disparities from the gradient. We chose to implement the disparity estimator presented in [8] because it does not use complex mathematical operations, such as trigonometric functions or roots, which require a more complex hardware implementation. The disparity estimator to be implemented is shown in Equation (4). Taking into account that we use a light field of 3×3 views, the summations in *P* disappear in the angular dimension, because we only have partial derivatives of the central image. We also omitted the summation in the spatial dimensions.

**Figure 10:**
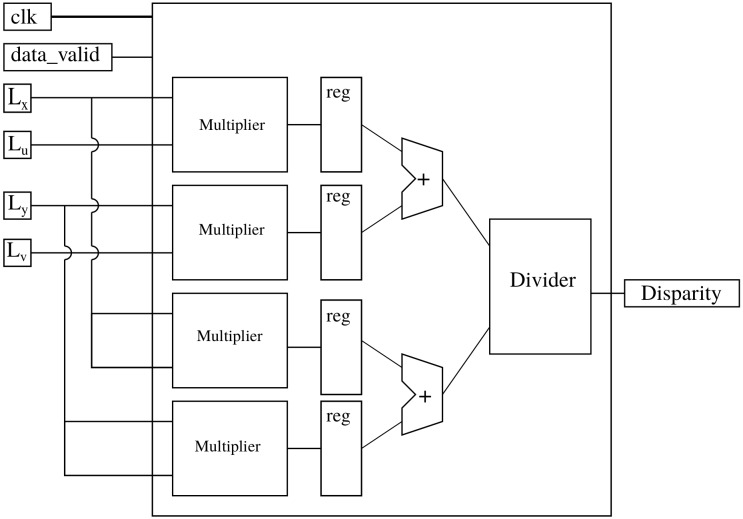
Block diagram of disparity estimation module.

Figure 10 shows a block diagram for the disparity computation. We use four parallel multipliers, two adders and one dedicated divider. The divider is designed with a pipeline architecture to achieve one result per clock cycle. After a latency period related to the length of pipeline, a result is obtained every clock cycle.

## 3. Experiments and Testing

### 3.1. Material

The implementation of hardware accelerators was done using the ZYBO development board [73]. The board includes a Zynq Z-7010 FPGA that is integrated with a dual-core ARM Cortex A9 processor. Although an accelerator is already implemented in hardware, the processor will be used for testing purposes. The processor runs a Petalinux operating system [74] and can directly communicate with the developed hardware over different system buses. The hardware development was done using VHDL language and VIVADO 14.4 [75].

### 3.2. Testing Methods

We employed two testing methods during the hardware implementation: simulation and actual execution. In the first method each design was tested using a testbench that read light-field data from a file and provided it to simulated hardware design. This allowed us to obtain the quantitative data shown in Section 4. Simulations were carried out with the ISIM included in ISE design suite 14.7 [76]. Using this procedure, we numerically compared the processing results and obtained a precise run-time estimation. In the second test phase, the design was implemented in real hardware and connected to the ARM processor embedded in Zynq. We ran a program on the processor to gather the light-field data over a TCP/IP socket from the PC and then send it to the implemented hardware accelerator. The results were read back by the ARM and sent to the PC for evaluation.

### 3.3. Test Data

To check the proper functioning of the developed designs, we generated several synthetic light fields using Blender [77] such that each light field was accompanied by the corresponding disparity ground truth. To do so, we used our own customised Blender plug-in.

**Figure 11:**
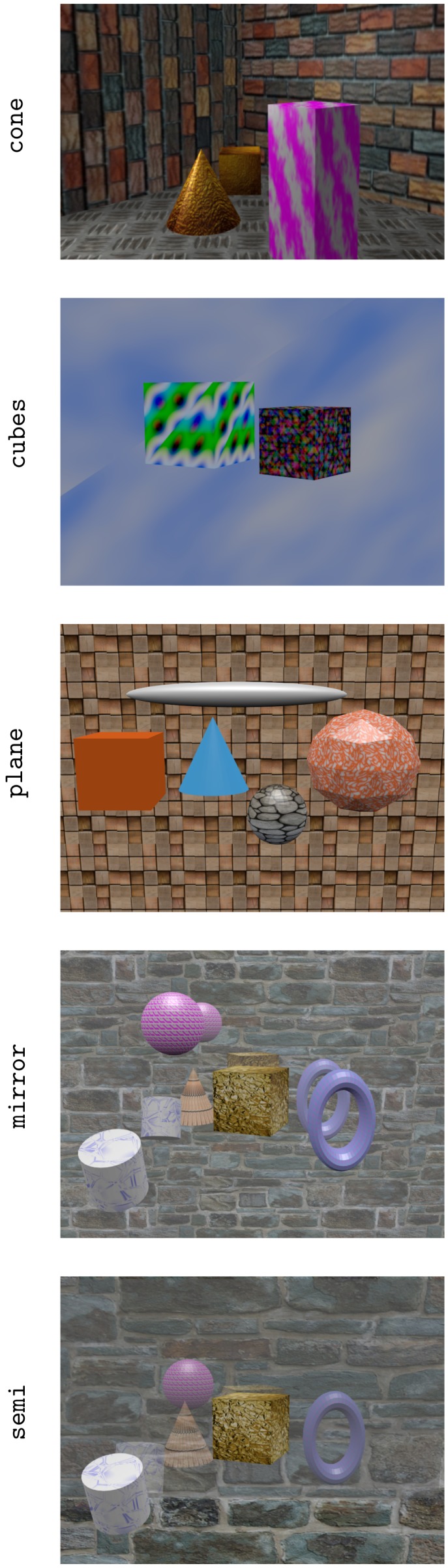
Central images of synthetic test light fields.

As we focused on local algorithms, to ensure the disparity estimation algorithm works, the disparity range was constrained as much as possible to the range of [−1,1] pixels/view. This enforces to situate the scene between a minimum distance and a maximum distance corresponding to the disparity limits. Following this constraint, a set of light fields with corresponding ground truths was created. Each light field comprised 3×3 views with a distance of 0.1m between them. The sensor size was 32mm. Table 4 shows the size of each view and the scenes’ disparity range. Please note that some parts of the scene fall out of the specified disparity range to test the effect on accuracy. In Figure 11, the central images of the synthetic light fields can be observed. Five scenes were considered. The cone scene is a scene with its disparity range slightly out of the algorithm’s working range. The cubes scene falls within the range. The plane scene again falls out. Finally, the mirror and semi scenes have some reflective elements included. This allowed us to test the algorithm’s robustness against such situations.

**Figure 12:**
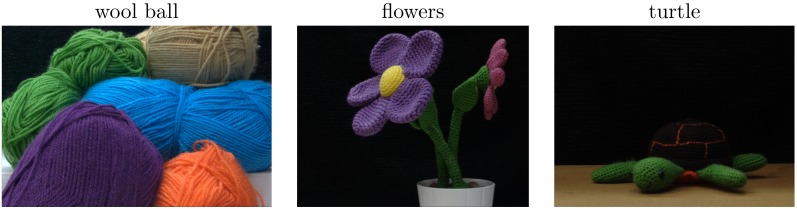
Central images of actual test light fields.

We also used a set of real light fields acquired via temporal multiplexing. The images were captured with a Nikon D-60 camera with a AF-S Nikkor 18–55 mm 1:3.5–5.6G lens. The displacement between viewpoints is 500 μm. The raw images have 3872×2592 pixels. However, the acquired images were cropped and downsized, obtaining three light fields with different shapes as specified in Table 5. The central images these light fields are shown in Figure 12.

## 4. Results

### 4.1. Logical Resources and Execution Times

Table 6 shows the resources needed to implement the two design alternatives considered in Section 2 to process a light field of 99×99×3×3. The designs require the number of view points to be $N_{u}\times N_{v}=3\times 3$. The spatial resolutions may vary. A change in the spatial dimensions, $N_{x}$ and $N_{y}$, does not significantly affect the resources required. The maximum achievable clock frequency is 102 MHz for the serial design and 38 MHz for the parallel.

Both designs were able to generate gradients and the disparity estimations during data input. Table 7 shows the achievable frame rates as a function of the light-field shape to be processed. We also show the frame rates achieved for a C++ implementation running a Intel^®^ Core ™i3-4130 CPU at 3.40 GHz. That implementation was done on a GNU/Linux using a single thread and compiled with code optimisation (-O3). Furthermore, the convolution operation was implemented taking advantage of the filters’ separability property (this was the optimal way to implement the operation). A second test was run on the embedded processor on a Raspberry Pi3 Model B+ board, which includes a Broadcom BCM2837B0 Cortex-A53 64-bit SoC with a clock frequency of 1.4 GHz [78].

### 4.2. Gradient Estimation

As all the operations in our two designs used fixed-point arithmetic, we compared the difference in the results of the designs via a reference implementation on a computer that uses floating-point arithmetic. Table 8 shows the mean absolute differences with respect to the floating-point implementation for the serial input design and Table 9 for the view parallel input design. An average per derivative was also computed, as well as the global average of all derivatives. It must be noted that the errors shown are for images with pixel values between 0 and 255. Although the error of the parallel input design is greater than the error with serial input, in percentage terms the difference is not significant.

### 4.3. Disparity Estimation

**Figure 13:**
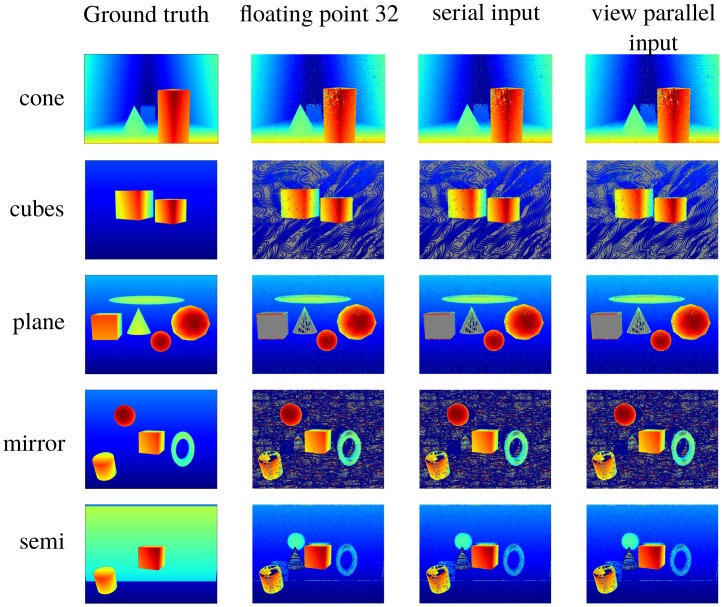
Disparity maps for the test images. Column 1: Ground truth. Column 2: Results with a floating-point implementation. Column 3: Results with serial input hardware. Column 4: Results with parallel input hardware. Warm colours are objects close to camera, while cold colours are objects far from camera. Gray pixels are positions where the algorithm did not provide a valid result.

This section presents the disparity results obtained using the output adaptor described above. As we noted in Section 3, we used Blender to generate the light fields we used as references. Figure 13 shows the disparity maps for each light field. The first column shows the ground truth, the second shows the result obtained with a floating-point implementation on a PC and third and fourth columns show the results obtained with hardware accelerators for serial input and view parallel input, respectively.

To provide quantitative insight, Table 10 shows the mean absolute differences between ground truth and a floating-point implementation, the serial input accelerator and the view parallel input accelerator. As can be seen, the second column shows greater errors than the first one and the third shows greater errors than the second one due to the effect of fixed point arithmetic.

**Figure 14:**
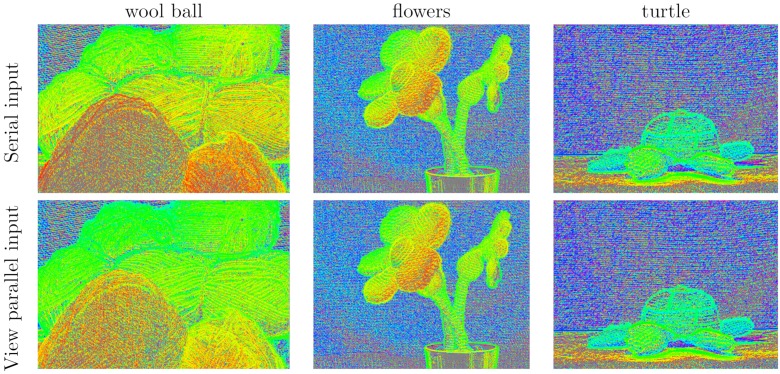
Disparity maps obtained with real light-field data. Row 1: Results with serial input hardware. Row 2: Results with parallel input hardware. Warm colours are objects close to camera while cold colours are objects far from camera. Gray pixels are positions where the algorithm did not provide a valid result.

While the synthetic data might provide some insight on the accuracy because a ground truth is available, it does not consider the effects that can appear in real data. For this reason, we conducted some tests on real light-field data. Figure 14 shows the results obtained with both designs for the light fields shown in Figure 12 and described in Section 3

It must be noted that the algorithm cannot provide a result in texture-less regions because divisions by zero arise in Equation (4). These pixels are marked with gray values in Figure 13 and Figure 14. They are also excluded from the mean absolute differences in Table 10.

## 5. Discussion and Conclusions

In this paper, we first compared the resources that both designs consume on the FPGA. The results show that both designs use a similar amount of resources, except in the case of DSP48E1s. This difference arises because with the view parallel input, the amount of data to be computed in parallel increases in the first stage of the processing pipeline and this requires more logic.

Regarding the execution times shown in Table 7, it is clear that the input format plays an important role because the view parallel input design allows one to achieve more than 28 fps for all tested image sizes, while the serial input produces just 8 fps for a resolution of 1280×1024 pixels. The table also shows that the serial design’s achievable frame rates are comparable to those achieved running the algorithm on a PC CPU. As can be observed, the performance is similar. However, if the comparison is done using an embedded processor, the results show that a speed-up of 7.5 was achieved with the serial input design and a speed-up considerably higher for the view parallel input design. None of the tested light-field shapes achieved real-time performance on the embedded processor.

The next step in our analysis consisted of testing the influence of using fixed-point arithmetic in the calculation. The derivatives were calculated using floating-point arithmetic on the computer. Then the same derivatives were computed for both processing architectures. Table 9 shows the mean absolute differences for the derivatives with respect to the floating-point implementation. It can be observed that average error introduced is below the quantization level of the images. As pointed out by Farid and Simoncelli in [72], the calculated derivatives suffer from systematic errors due to the use of truncated derivative filters and random errors due to image noise.

Table 10 shows the mean absolute error of a floating-point implementation with the disparity ground truth. It also shows these errors for both proposed architectures. The differences begin at second or third fractional digit. This means that errors increase less than 0.01 pixels/view which we do not consider significant. The conclusion is that errors are mainly due to the algorithm’s inherent limitations and not to implementation. In [79], an analysis of the influence of systematic errors and random errors in the final disparity estimation is provided. Here, an additional error source is introduced because of the truncation in fixed-point arithmetic. To know the effect of truncation requires further analysis because it depends on the concrete implementation. Another point that might be considered is the optimisation of the filter coefficients taking into account the limitations in numeric precision, but this study falls outside the scope of this paper.

Figure 13 compares the results obtained with a floating-point implementation on a computer to the results obtained with both input designs. No significant differences can be observed. We also tested both designs with the set of real light fields described in Section 3. The results of these tests are shown in Figure 14. Again, there is no significant difference between the results for the two designs. Furthermore, the results show that one can apply the designed system to real data, as we expected. These results could be further improved by fusing multiple depth maps or applying a refinement technique in a later processing stage, as in [42].

We also want to highlight the differences between our designs and other depth from light field approaches published in the literature. Table 11 compares some features of our proposed designs to other approaches. The approach presented in [70] shows an implementation on FPGA of the belief propagation algorithm used in stereo systems. The main drawback with that approach is that a $N_{x}\times N_{y}\times K$ cost volume must be generated to extract the optimal value between *K* candidate values at each of the $N_{x}\times N_{y}$ pixels of the output disparity map. The generation of the cost volume is not addressed in the paper. Furthermore, the design requires the storage of the volume in the on-chip memory of the FPGA, which could be an important limitation when high resolutions are desired. Regarding processing times, it must be remembered that the belief propagation is an iterative process, so the processing time would depend on the number of iterations that must be carried out. According to the time analysis presented in [70], the number of clock cycles to refine one output is $\frac{W\times H}{2}\times N\_\mathrm{iter}$. Both of our proposed designs show better processing times. Furthermore, our designs also require less internal memory. On the other hand, with the proposed designs, the outputs are calculated at same time as the input is read in, and the result is produced only a few clock cycles after read-in is finished. The design proposed in [71] allows one to output a $H_{out}\times W_{out}$ disparity map with *K* disparity levels. Here an off-chip memory was used and the authors proposed an adequate caching scheme that uses on-chip memory but do not specify the concrete dependency from the input data shape. This design needs time to transfer the data to memory and then it performs processing. This is not the case for the our proposed designs, where the output is generated simultaneously with the data read-in. We have also calculated the achievable frame rates to obtain a disparity map of 640×480 pixels with a clock frequency of 100 MHz, which seems to be a realistic assumption for all the designs except the view parallel. The results show that the proposed designs outperform the others described here and use less on-chip memory. From a qualitative point of view, it must also be highlighted that the proposed designs are not limited by the number of disparity levels because the algorithm does not consider a cost volume. Instead, they make the calculation continuous while restricting it to a short disparity range.

The current implementations have some limitations that must be pointed out. Currently, the number of views is fixed to 3×3. However, an extension to more views would be straightforward and would not likely require a significant increase in the use of logical resources. Depending on the shape of the light field, more memory blocks could be needed. In our designs, the derivative filter size is also fixed to 3×3×3×3, which is the minimum. If a greater filter size is desired, more basic components must be added to the grid. However, we think it would be more interesting as an alternative to increasing filter size to add the averaging operation shown in Equation (4). Another alternative would be to generate multiple disparity maps that have to be fused in a later stage, as proposed in [42].

In conclusion, we presented two architectures that share a common construction block and make it possible to obtain a disparity map nearly simultaneously with the camera read-out. The view parallel architecture seems to be more attractive since it allows one to achieve a higher degree of parallelism and increases the performance to real time, even for high resolution images, on a low-range FPGA. Compared to other designs, both proposed architectures seem to outperform others when operating at the same clock rate. The performance of the serial input design is very similar to that of a conventional CPU, while the view parallel design clearly outperforms it. The main advantage of both architectures is that they can be integrated into an embedded processing system based on SoC in which a processor and an FPGA are available. If the goal is to achieve real-time performance on an embedded system, the algorithm should be implemented on FPGA or other processing alternatives, such as mobile GPUs. Direct implementation on embedded processors seems not to be a suitable alternative. The implementation on FPGA also allows integration with other subsystems, such as capture or decoding and rectification. The produced output could also be fed into other subsystems implemented on the same FPGA.

## Figures and Tables

**Table 1:** Non-zero and distinct positive filter coefficients for serial input.

Coeff.	Value
$W_{0}$	p[0]p[0]p[0]d[1]
$W_{1}$	p[1]p[0]p[0]d[1]
$W_{2}$	p[1]p[1]p[0]d[1]
$W_{3}$	p[1]p[1]p[1]d[1]

**Table 2:** Non-zero and positive distinct filter coefficients for parallel input.

Coeff.	Value
$W_{0}$	p[0]d[1]
$W_{1}$	p[1]d[1]
$W_{2}$	p[0]p[0]
$W_{3}$	p[0]p[1]
$W_{4}$	p[1]p[1]

**Table 3:** Description of I/O signals of basic component.

Signal	Function
clock	System clock, block receives one datum at each rising edge.
reset	Restarts the system.
data_in	Pixel × coefficient, after selection based on position and clock cycle.
mem_reg	Indicates if data_in must be added to the value stored in the register or the value read from memory.
wr_en	Indicates that the block must write data to memory.
rd_en	Enables reading from memory. If this signal is low, the memory output is zero.
rd_addr	Read address
wr_addr	Write address (one less than rd_addr)
data_out	Output data

**Table 4:** Summary table of light-field generated.

Name	Size	Disparity		Focal Length
		Min.	Max.	
cone	300×198	−1.77	1.33	35 mm
cubes	798×600	−0.98	0.95	50 mm
plane	1023×768	−1.36	1.21	35 mm
mirror	798×600	−1.22	1.19	35 mm
semi	798×600	−1.25	1.03	35 mm

**Table 5:** Actual light-field data set.

Light Field	Shape
wool ball	648×435×3×3
flowers	726×567×3×3
turtle	534×402×3×3

**Table 6:** Comparison of resources employed.

Resource	Serial Input		Parallel Input	
	#	%	#	%
Slice registers	2436	6%	3386	9%
Slice LUTs	6086	34%	6227	35%
LUT-FF pairs	1235	16%	1675	21%
BUFG/BUFGCTRLs	1	3%	1	3%
BlockRam	36	60%	36	60%
DSP48E1s	8	10%	58	72%

**Table 7:** Frame rates for serial and parallel input designs.

Light-Field Shape	CPU (fps)	Embedded CPU (fps)	Proposed Serial Input (fps)	Proposed Parallel Input (fps)
640×480×3×3	28.46	4.55	36.89	123.7
800×600×3×3	18.69	2.89	23.61	79.17
1024×768×3×3	11.65	2.06	14.41	48.32
1366×768×3×3	8.77	1.50	10.8	36.22
1280×1024×3×3	7.00	1.15	8.65	28.99

**Table 8:** Mean absolute differences of serial input design with respect to reference implementation using floating-point arithmetic. Pixel values range from 0 to 255.

	$L_{x}$	$L_{y}$	$L_{u}$	$L_{v}$
cone	0.948532	0.263783	0.892295	0.241141
cubes	0.172564	0.077751	0.431636	0.046296
plane	1.330916	0.276368	0.684091	0.260979
mirror	0.975316	0.773819	1.721000	1.059183
semi	0.679706	0.379349	0.488751	0.355860
**AVERAGE**	0.8214068	0.354214	0.8435546	0.3926918
	0.602967			

**Table 9:** Mean absolute differences of parallel input design with respect to reference implementation using floating-point. Pixel values range from 0 to 255.

	$L_{x}$	$L_{y}$	$L_{u}$	$L_{v}$
cone	0.949253	0.264179	0.892603	0.241443
cubes	0.172618	0.077776	0.431746	0.046305
plane	1.331450	0.545600	2.070984	0.491962
mirror	0.975436	0.773956	1.721910	1.059512
semi	0.680080	0.379738	0.488941	0.356212
**AVERAGE**	0.821767	0.408250	1.121237	0.439087
	0.697585			

**Table 10:** Mean absolute differences from ground truth. Disparity ranges for each scene are shown in Table 4.

Scene	F.P.	Serial	V. Parallel
cone	0.16977168	0.17220395	0.18224397
cubes	0.08242574	0.08350541	0.08484645
plane	0.11949781	0.12752187	0.12754149
mirror	2.36933125	2.36754665	2.37261304
semi	0.52248116	0.52360019	0.52364982

**Table 11:** Comparison with other depth from light field implementations on FPGA.

	Proposed Serial Design	Proposed View Parallel Design	Magdaleno et al. [70]	Chang et al. [71]
Output size	$N_{x}\times N_{y}$	$N_{x}\times N_{y}$	$N_{x}\times N_{y}$	$H_{out}\times W_{out}$
Colour channels	Intensity	Intensity	–	Intensity
Processing time (clock cycles)	$N_{x}\times N_{y}\times N_{uv}\times N_{uv}$	$N_{x}\times N_{y}$	$\frac{N_{x}\times N_{y}}{2}\times \left(N\_\mathrm{iter}+1\right)$	$K\times H_{out}\times W_{out}+3$
On-chip memory	$4\times 3\times 3\times \left(N_{uv}/3\right)\times N_{x}$	$4\times 3\times 3\times \left(N_{uv}/3\right)\times N_{x}$	$\propto K\times N_{x}\times N_{y}$	Not specified
Off-chip memory	Not used	Not used	Not used	Required
Delay (clock cyles)	5	9	$N_{x}\times N_{y}\times K$	$N_{uv}\times 3\times N_{uv}\times N_{x}\times N_{y}$
Frame rate (fps) @ 100 MHz clock and output size: 640×480	~36	∼347 (@38 MHz 123 fps)	~25 (N_iter=25)	~27 (K=12)
